# PHDs inhibitor DMOG promotes the vascularization process in the AV loop by HIF-1a up-regulation and the preliminary discussion on its kinetics in rat

**DOI:** 10.1186/s12896-014-0112-x

**Published:** 2014-12-28

**Authors:** Quan Yuan, Oliver Bleiziffer, Anja M Boos, Jiaming Sun, Andreas Brandl, Justus P Beier, Andreas Arkudas, Marweh Schmitz, Ulrich Kneser, Raymund E Horch

**Affiliations:** Department of Plastic and Hand Surgery, University Hospital of Erlangen, Friedrich Alexander University, Erlangen Nuernberg, (FAU) Germany; Department of Plastic Surgery, Union Hospital, Huazhong University of Science and Technology, Wuhan, China; Department of Hand, Plastic and Reconstructive Surgery, Burn Care Unit, BG-Trauma Centre Ludwigshafen, University of Heidelberg, Ludwigshafen, Germany

**Keywords:** Hypoxia, Vasculorization, Arteriovenous loop, PHD inhibitor, Dimethyloxallyl Glycine, Kinetics

## Abstract

**Background:**

The Arterovenous Loop (AV Loop) model is a vascularization model in tissue engineering research, which is capable of generating a three dimensional *in vivo* unit with cells as well as the supporting vessels within an isolation chmaber. In our previous studies the AV loop in the isolation chamber was discovered to undergo hypoxia, characterized by Hypoxia Inducible Factor (HIF) up-regulation. The vascularization followed the increase of HIF-α temporally, while it was spatially positively correlated with the HIF-α level, as well. This study aims to prove that HIF-1a up-regulation is the stimulus for vascularization in the AV loop model.

**Method:**

The AV loop model in rats was created by interposing a femoral vein graft into the distal ends of the contralateral femoral artery and vein, and the loop was embeded in fibrin matrix and fixed in isolation chamber. PHD (prolyl hydroxylases) inhibitor DMOG (Dimethyloxallyl Glycine) was applied systemically in the rats in 40 mg/KG at day 0 and day 3 (DMOG-1), or in 15 mg/KG at day 8, day10 and day12 (DMOG-2). Two weeks later the specimens were explanted and underwent morphological and molecular evaluations.

**Results:**

Compared to the control group, in the DMOG-2 group the HIF-1α positive rate was siginicantly raised as shown in immunohistochemistry staining, accompanied with a smaller cross section area and greater vessel density, and a HIF-1α accumulation in the kidney. The mRNA of HIF-1α and its angiogenic target gene all increased in different extends. Ki67 IHC demostrate more positive cells. There were no significant change in the DMOG-1 group.

**Conclusion:**

By applying DMOG systemically, HIF-1α was up-regulated at the protein level and at the mRNA level, acompanied with angiogenic target gene up-regulateion, and the vascularization was promoted correspondingly. DMOG given at lower dosage constantly after one week tends to have better effect than the group given at larger dosage in the early stage in this model, and promotes cell proliferation, as evidenced by Ki67 IHC. Thus, this study proves that HIF-1a up-regulation is the stimulus for vascularization in the AV loop model and that the process of the vessel outgrowth can be controlled in the AV Loop model utilizing this mechanism.

**Electronic supplementary material:**

The online version of this article (doi:10.1186/s12896-014-0112-x) contains supplementary material, which is available to authorized users.

## Background

Tissue engineering is a very promising technology but until now has a limitation of obtaining large three dimensional constructs with a reliable vascularization that would resemble living tissue. In contrast to *in vitro* models, in *in vivo* tissue engineering studies, the cells planted into the three-dimensional construct require a sufficient perfusion from local blood capillary network [1]. As the basic units that built up the organism, in most cases, the cells draw their nutrition from the diffused molecules from the blood capillaries that are no more than 200 μm away [2,3]. Thus, the key to successful tissue construction is to vascularize the constructs quickly and efficiently.

The Arteriovenous Loop (AV Loop) model first described in 1979 [4] is an *in vivo* vascularization model for surgical purposes. It is composed by interposing a femoral vein graft into the distal ends of the contralateral femoral artery and vein, and then the loop is embedded by specific matrixes within an isolation chamber, where an axial vascularization is generated [5]. By manipulating the matrix and the chamber that hold the loop, the model has been applied in various tissue engineering studies. Lokmic et al investigated the spontaneous microcirculatory development of the AV loop in a protected space within a polycarbonate chamber, suggesting that the model possessed enormous potential by manipulating the chamber structure to conduct the growth of the tissue to required directions [6]. The AV loop has also been applied for bone tissue engineering with porous solid matrices [7,8]. The model could also carry cells suspended in soft matrix and provide an in vivo culture platform thus possesses the potential to generate a target organoid eventually. Brown et al seeded islet cells with matrigel into the model and the cells survived 3 weeks after implantation [9]. In another study on muscle tissue engineering using the AV loop model, primary myoblasts were seeded and later individual myoblasts fused to form multinucleated myotubes [10].

The AV-loop model provides a highly vascular, isolated tool for in vivo tissue engineering as well as angiogenesis and anti – angiogenesis studies. The angiogenic property is driven by different forces. Mechanical forces play themselves an important role through shear stress in producing a subcritical ischemic area where angiogenesis and vasculogenesis must start. So angiogenesis and vasculogenesis can also be a physically (mechanically) induced phenomenon from effects of turbulences and shear stress [11].

The AV loop model is a protected space separated from the surrounding tissue, and therefore is also a hypoxic environment for the inner tissue and cells. Hence the AV loop implanted in the isolation chamber is also a hypoxic model [12-15]. Hypoxia Inducible Factor (HIF) is a transcriptional factor that is the master regulator of oxygen homeostasis and it also plays an essential role in angiogenesis [16-18]. HIF consists of α and β subunits. HIF-1α is one of the three isoforms of HIF-α, which is most critical for hypoxic response [19]. There are over 100 angiogenic genes found up to date that are directly or indirectly regulated by HIF-1α, such as Vascular Endothelial Growth Factor-A (VEGF-A) [20] and Basic Fibroblast Growth Factor (bFGF) [21].

The HIF-1α subunit is constantly expressed, but will be degraded rapidly under normoxic condition. The Hydroxylases, mainly the Prolyl hydroxylase (PHD), [22] are greatly involved in the degradation of HIF-1α. In well-oxygenated cells, the prolyl hydroxylation is catalyzed with the presence of Fe (II) and oxygen. Whereas in hypoxia when the PHD is blocked or inhibited due to lack of oxygen, HIF-1α is stabilized and dimerizes with the β subunit, binds to target genes, and leads to the downstream activations towards increase target gene transcription.

**Figure 1:**
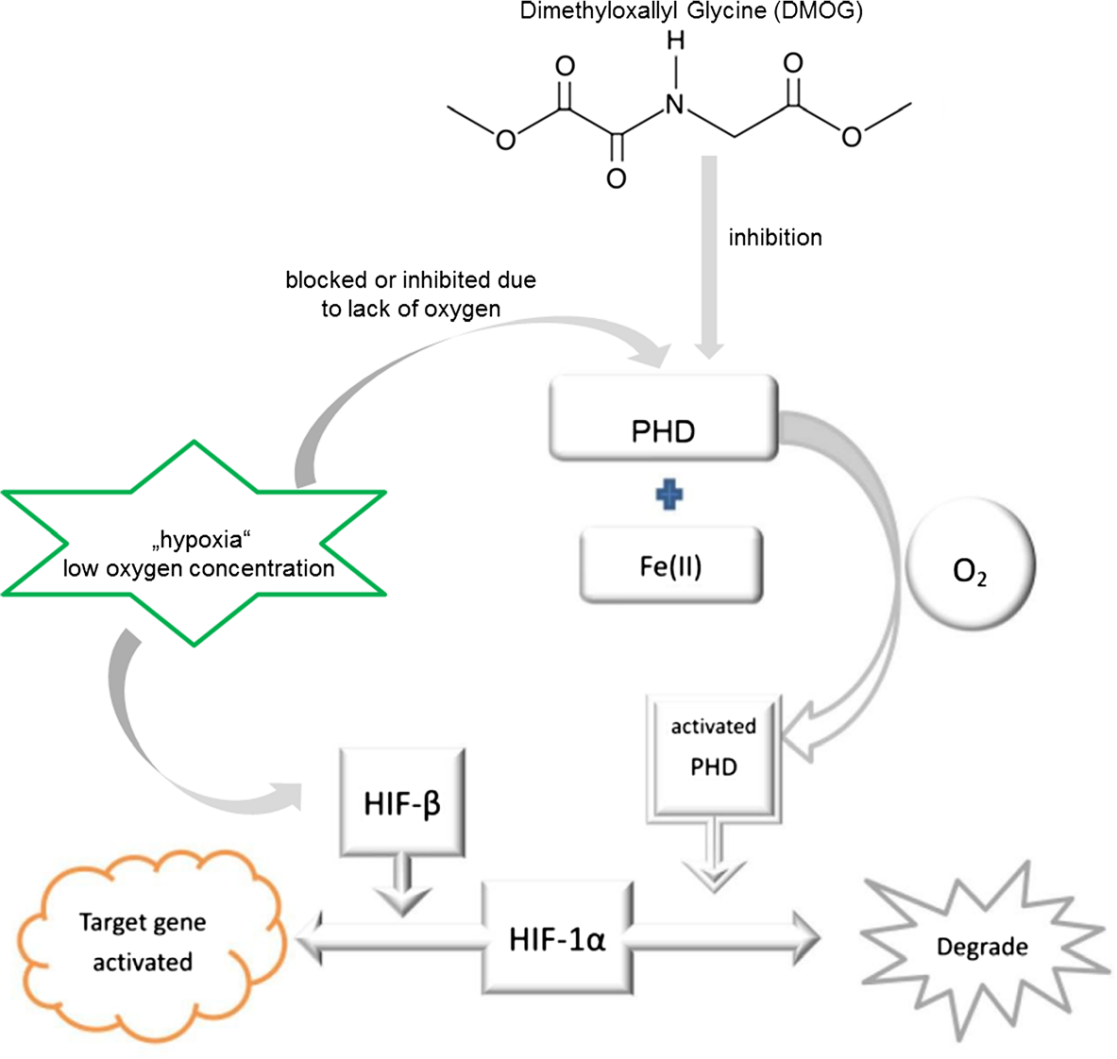
**The Hydroxylase pathway of HIF**-**1α and DMOG structure.** The Prolyl hydroxylase (PHD) is catalyzed with the presence of oxygen and Fe (II), then subsequently involved in the HIF protein degradation. Otherwise, HIF-1α is stabilized and dimerized with the HIF-β, and activates the target genes. DMOG structure (From SciFinder®).

The endogenous HIF-1α levels can be increased by the suppression of PHD activity, either by reducing the cellular oxygen level or by combining the Fe (II) competitively. Dimethyloxallyl Glycine (DMOG) is a cell permeable, competitive inhibitor of the PHDs [23]. DMOG is an analogue of 2-oxoglutarate, and in this way it inhibits not only the HIF prolyl but also asparaginyl hydroxylases. Beside that it is predicted to inhibit other members of 2-oxoglutarate-dependent dioxygenases. DMOG has been proved to be able to stabilize HIF-1α both in vitro and in vivo [24-27], which can be superior to adding limited kinds of growth factors [28] (Figure 1).

HIF-1α could accumulate in the nuclei of the hypoxic cells and the protein can be detected by immunohistochemistry staining. In our previous study, the loop sealed in the isolation chamber was discovered to undergo hypoxia, characterized by HIF-1α up-regulation [29]. The vascularization followed the increasing of HIF-1α temporally, while spatially it was positively correlated with the HIF-1α level, as well (Data not shown). Thus, to prove the hypothesis, the PHD inhibitor DMOG was applied to stablize the HIF-1α, to see weather the increase of HIF-1α would induce the increase of vascularization in the AV loop.

## Methods

### Animal model preparation

Syngenic male Lewis rats at 300 g (Charles River GmbH, Sulzfeld, Germany were used in the experiment. The rats were specific pathogen free (SPF), kept with optimized and standardized animal housing. Ethical rules have been followed during the whole research.

**Figure 2:**
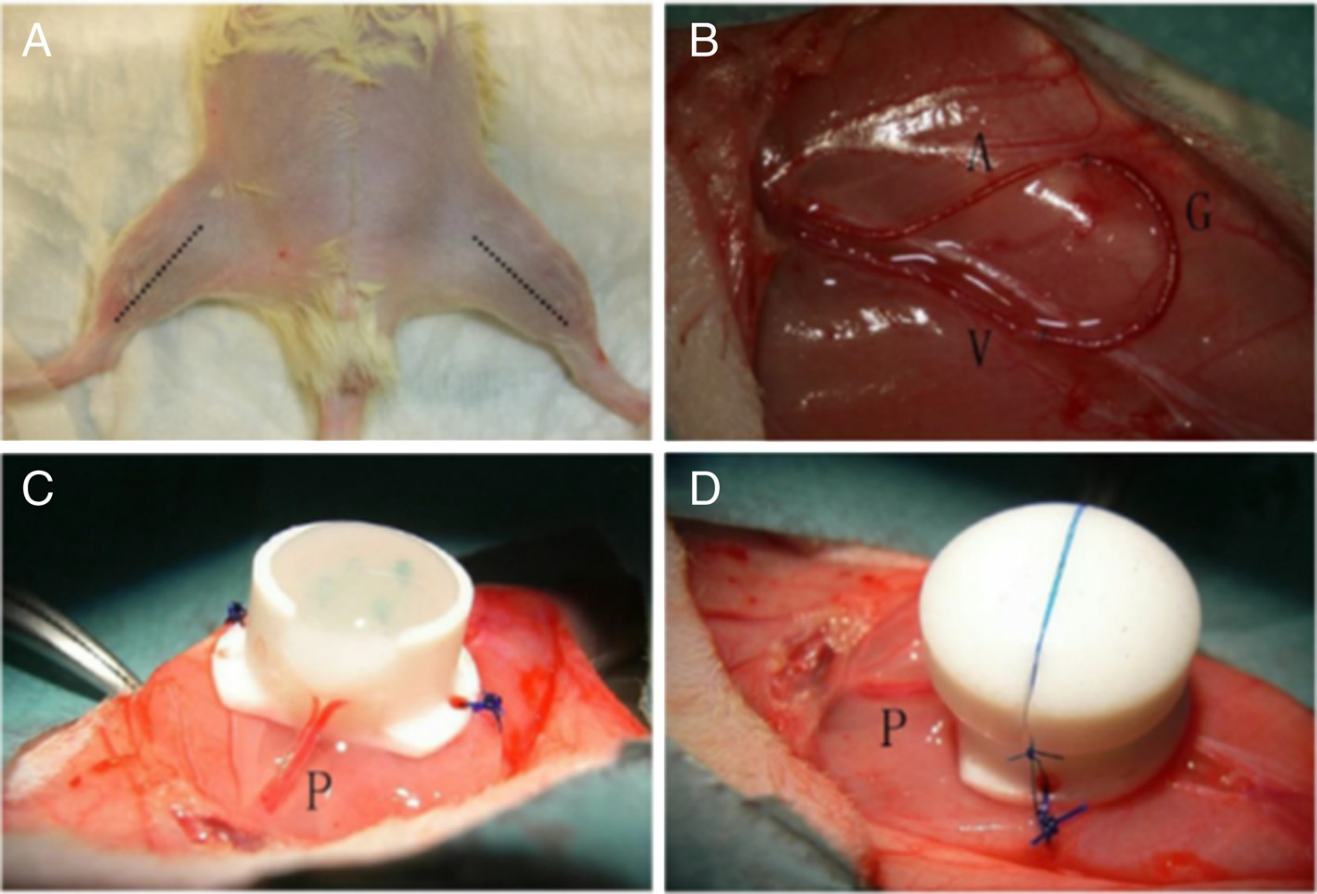
**The surgical procedures of AV Loop preparation.** **A)** Incisions on the thighs. **B)** The AV Loop: A = artery, V = vein, G = graft from the contralateral femoral vein. **C)** Loop fixed in the chamber, embedded by fibrin clot. **D)** The isolation chamber covered by the lid and fixed on the left thigh. P = pedicle.

All surgeries were performed under magnification with the same microscope (OPMI Vario 700, Karl Zeiss, Jena, Germany) by the same micro surgeon. The rat was laid on the back on the warming plate at 37°C under anaesthesia with 2% Isoflurane inhalation (Figure 2a). The left femoral vessels were exposed by the incision on the middle of the thigh, with the vasa vasorum removed and branches coagulated. The 1.5 cm vein graft was harvested from the contralateral femoral vein in the same way and was interposed between the distal ends of the left femoral artery and vein (Figure 2b). 11-0 suture (Ethicon, Norderstedt, Germany) was used to inosculate the end-to-end anastomosis. When the loop was accomplished, it was then placed in a cylindrical Teflon chamber, fixed by 4 stabs and embedded by 720 ul fibrin gel made up of 4 IU/ml fibrinogen and 2 IU/ml thrombin (Tisseel VH S/D, Baxter Healthcare S.A., Wallisellen, Switzerland) (Figure 2c). The chamber was sealed by a Teflon lid with 6-0 suture (Ethicon) and fixed on the thigh with 3-0 suture (Ethicon). (Figure 2d). After the skin closure, the rat was given 60 I.U. heparin subcutaneously and antibiotics (Veracin compositum) 0.2 ml intramuscularly. The wound was covered by Aluminum spray.

### Group settings and drug administration

The rats were randomly allocated into 3 groups:

1. *control group (n = 6),*
2. *DMOG-1group (n = 6): DMOG 40 mg/kg, i.p. at day 0 and day 2,*
3. *DMOG-2 group (n = 6): DMOG 15 mg/kg, i.p. at day 8, day 10 and day 12.*

DMOG (cayman, 71210) was freshly dissolved with saline before use. The animals underwent an observation period of 2 weeks. Upon the date for explantation, the distal 2/5 of the specimens were frozen in liquid nitrogen, stored in -70°C fridge before sent to further RNA isolation and qPCR analysis. The proximal 3/5 of the specimens was fixed in 4% PFA for routine histology analysis. Kidneys were also kept as organ control to see the DMOG remained systemically.

### Immunohistochemistry

#### ROI

**Figure 3:**
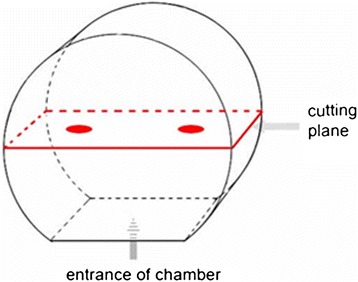
**The cutting plane in the fibrin clot containing a loop.** The plane in the picture demonstrated the slide cut in the middle of the clot. As the blade cut across the specimen, the transverse section was harvested, which was marked with red board.

Transverse section across the central was picked, (Figure 3). In each slide, the ROI were taken in 4 directions from the central vessels.

The plane in the picture demonstrated the slide cut in the middle of the clot. As the blade cut across the specimen, the transverse section was harvested, which was marked with red board.

### HIF-1α IHC

The staining was performed with Dako Catalyzed Signal Amplification (CSA) system (K1500) based on “ABC method”. After the 7 min cooking in pressure cooker at 121°C for antigen retrieval, The slides were successively blocked with Peroxidase Block for 10 min, Avidin Block (Vector Avidin/Biotin blocking Kit) for 20 min, Biotin block(Vector Avidin/Biotin blocking Kit) for 20 min and Protein Block (CSA kit) for 30 min. The slides were subsequently incubated with the primary antibody Polyclonal rabbit anti HIF-1α (Cayman 10006421) in 1:50000 dilution in 2 ~ 8°C fridge overnight, and then the secondary antibody Biotinylated polyclonal Goat anti Rabbit (Dako E0432) in 1:2000 dilution in 2% rat serum for 15 min in room temperature, succeeding the incubation of Strepavidin-Biotin Complex (SBC, from CSA kit, prepared 30 min before use), Amplification reagent (CSA kit) and Strepavidin-Peroxidase (CSA kit) for 15 min separately. In the end the slides were developed by DAB.

### Lectin IHC

Cross sections were cut from the central part of the Loop and Lectin IHC was performed. The newly grown vessels in the granulation tissue in the AV loop were Lectin IHC positive, appeared in brown lining in lumens. After the pictures of each slide were taken by camera with the Leica application software, the area of cross section was measured by Leica application software. The absolute vessel number was counted manually and by dividing it to the cross section area, the vessel density was calculated as well.

The slides were cooked in pressure cooker for 1 min for antigen retrieval. Isolectin B4 Biotinylated antibody (Bandeiraea Simplicifolia Sigma L2140) was diluted to 1:250 with Tris buffer, incubated in 2 ~ 8°C fridge overnight, following the reaction with Streptavidin-peroxidase for 30 min. The signal was developed by DAB for approximately 10 min.

### Ki67 IHC

After 1min cooking in high pressure cooker, the staining was performed with Polymer kit (ZytoChem Plus AP Polymer Kit), using the antibody Monoclonal rabbit anti Ki67 (Zytomed RBK 027) in 1:200 dilution, and developed by Fast red.

Generally, unless specifically stated, the slides were incubated horizontally at room temperature (20°C). After the incubations there were three times of bath in Tris-tween buffer, only the rinse after the blocking applied Tris buffer without tween.

### Real-time Polymerase chain reaction (PCR)

RNA was isolated with the RNeasy® Fibrous Tissue Midi kit (Qiagen, Hilden, Germany) according to manufacturer’s instructions. cDNA was synthesized using 500 ng total RNA with random hexamer priming and the RevertAid™ First Strand cDNA Synthesis Kit (Thermo Scientific, Inc., Waltham, MA, USA). Real time PCR was performed in a CFX96 Touch™ Real-Time PCR Detection System and analyzed with the CFX Manager™ Software (Bio-Rad Laboratories, Inc., Hercules, CA, USA). The 20 μl total reaction volume in each well of 96-well plate included: 10 μl SsoAdvanced™ SYBR® Green Supermix, 0.6μl forward primer, 0.6 μl reverse primer, 7.8 μl nuclease-free water, and 1 μl cDNA (25 ng). The amount of each transcript was normalized to GAPDH. The mRNA expression of the control loop served as control and was set as 1. The calculation of the expression ratios was performed using the comparative Ct-method. Following is the list for the primers that were applied: Primer (5’-3’):

- r_eNOS_forward: TGCCACCTGATCCTAACTTGCCTT,
- r_eNOS_reverse: ATCGGCAGCCAAACACCAAAGT;
- r_Flt-1_ forward: ACAGCAATGTGTTCCACAGCGT,
- r_Flt-1_reverse: TGGTTTCCTGCACCTGTTGCTT;
- r_KDR_ forward: AATGCATTGTGCTGGCTGTGGT,
- r_KDR_reverse: AACCGTGAGAGCAAGCAACCTT;
- r_VE-Cadherin_ forward: CGCTAACAGTGGCTGTGTGCAAAT,
- r_VE-Cadherin_reverse: ACAGAAAGATGGCTACCAGTGCCT;
- r_vWF_ forward: TGAACGCAAGTGTCTGGCTGAA,
- r_vWF_reverse: TGCACCTTGGCTGTGATGTCTT;
- r_VEGF_ forward: AATGATGAAGCCCTGGAGTG,
- r_VEGF_rev_RT: ATGCTGCAGGAAGCTCATCT;
- r_HIF-1α_ forward: TGCTTGGTGCTGATTTGTGA,
- r_HIF-1α_reverse: GGTCAGATGATCAGAGTCCA;
- r_GAPDH_ forward: AATGCATCCTGCACCACCAACT,
- r_GAPDH_reverse: ATCCACAGTCTTCTGAGTGGCAGT.

### Evaluation and statistics

Pictures for the ROI were taken by the same microscope (Zeiss Carl Zeiss Meditec Vertriebsgesellschaft mbH, Oberkochen, Germany). The measures of the area of the clot were made by the software Leica Application (Leica Microsystems, Wetzlar, Germany). The counting of the vessels and cells in each ROI was made manually by blind tester. Statistical analysis was performed using Student’s-Test and with SPSS. P < 0.05 was considered of statistical significance and labeled.

### Research involving human subjects

There was no Research involving human subjects (including human material or human data) that is reported in the manuscript.

All experiments were approved by the animal care committee of the University of Erlangen and the Government of Mittelfranken, Germany (54-2532.1-28/09).

## Results: (whole section was reorganized)

### Systemic and local effects of DMOG: HIF-1α is positive in DMOG-2 group in kidneys and HIF-1α positive cells increased significantly in DMOG-2 group in the AV loop

**Figure 4:**
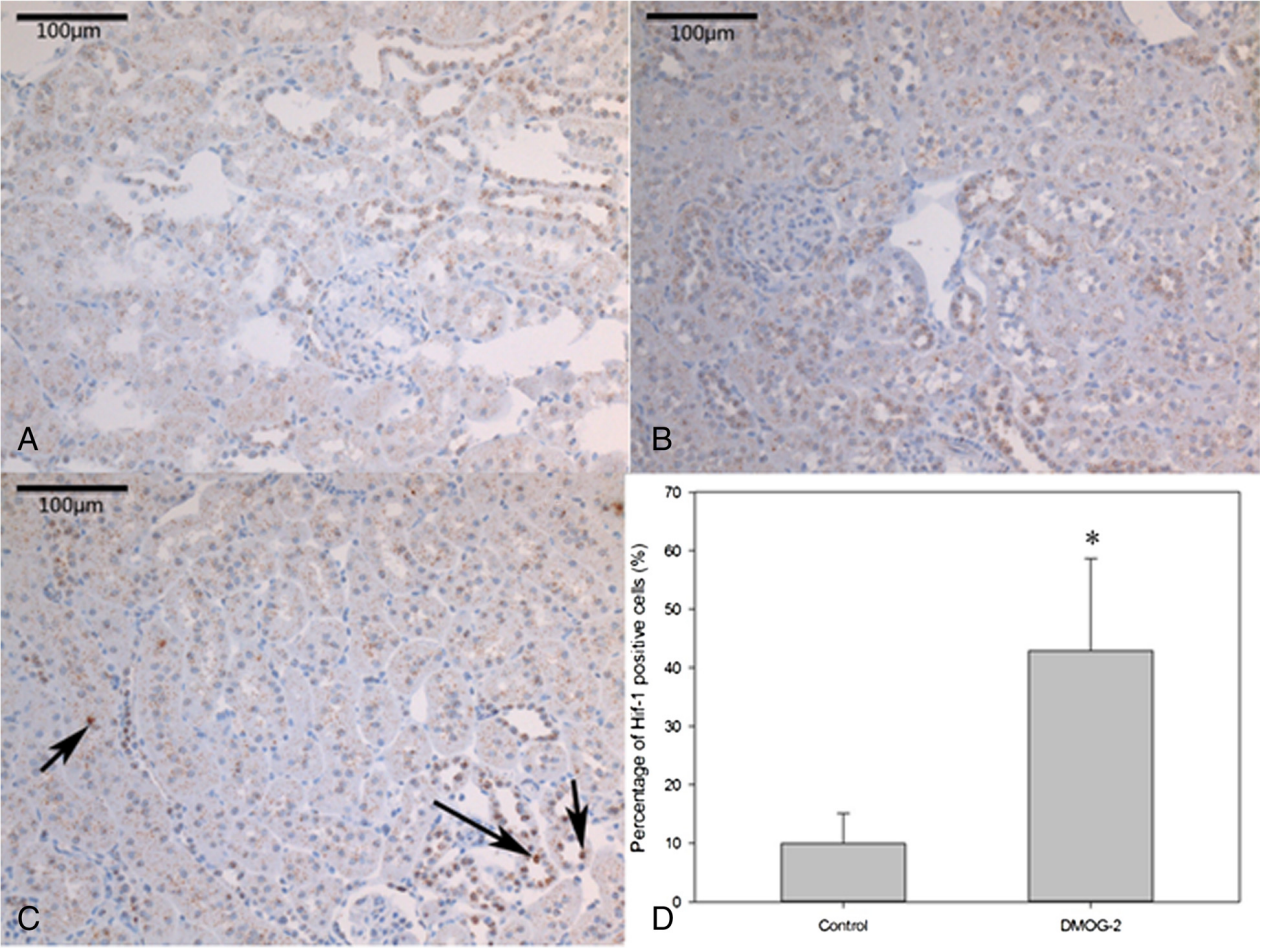
**HIF-** **1α IHC for kidney** **(×200)** **and Percentage of HIF**-**1α positive cells in the AV loop.** The HIF-1α IHC for kidneys taken under ×200 magnification. In control group **(A)** and DMOG-1 group **(B)** the kidneys were negative. In DMOG-2 group **(C)** HIF-1α positive cells were intensive brown in nuclei (pointed by arrows). **(D)** The slides from the central part of the Loop was cut and stained for HIF-1α IHC. In each slide 8 ROIs were taken from the up, down, left and right direction to the central artery or vein under x400 magnification. The brown nuclei cells recognized as HIF-1α positive cells and the blue nuclei cells recognized as HIF-1α negative cells, so the percentage of percentage of HIF-1α positive cells could be calculated. The DMOG-2 group that received DMOG i.p. injection in 15mg/KG at day 8, day10 and day12 had a higher HIF-1α positive rate than the control group. (48% versus 10% , *p < 0.05).

The effect of the systemic application of DMOG can only be shown in DMOG-2 group. As HIF-1α accumulated in the nuclei in hypoxic cells, the positive signal appeared in intensive brown in IHC staining. In DMOG-1 group (DMOG given at day 0 and day 2 at 40 mg/Kg) HIF-1α IHC was negative at day 14. However, in DMOG-2 group (DMOG given at day 8, 10, and 12 at 15 mg/Kg) a few HIF-1α positive cells in the kidney can be observed (Figure 4 A,B,C).

Beside systemically effects of DMOG HIF-1α positive cells increased significantly in DMOG-2 group in the AV loop (Figure 4D).

8 ROIs were taken in each slide, in 4 directions from the central artery and vein. In each picture, HIF-1α positive cell number and total cell number were counted manually and the percentage of HIF-1α cells was calculated. Comparing to the control group, the DMOG-2 group had 48% HIF-1α positive cells, which is significantly higher than the control group that had 10% HIF-1α positive cells out of the total cell ingredients (* P < 0.05).

### DMOG effects on the vascularisation in the AV loop: higher vessel density and smaller cross section area in DMOG-2 group

**Figure 5:**
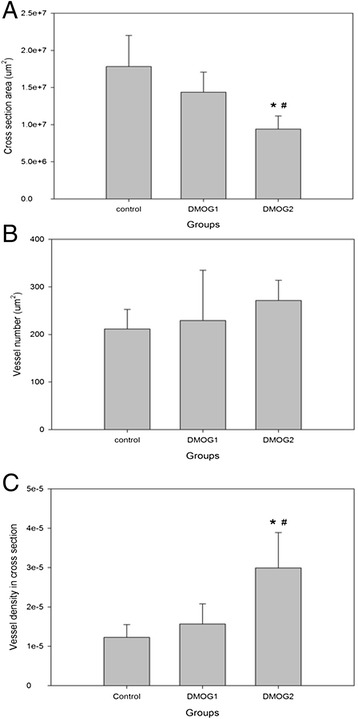
**Cross section area/** **absolute vessel number/** **vessel density in cross section.** Cross section was cut from the central part of the Loop and Lectin IHC was performed. The cross section area was measured by Leica application software **A)**. **Cross section area** (**μm** ^**2**^): the DMOG-1 group (14.4 x10^6^μm^2^) tended to be smaller than the control group (17.8 x 10^6^μm^2^) and bigger than the DMOG-2 group (9.4 x 10^6^μm^2^), but no statistic significance was found. The DMOG-2 group shrinks significantly comparing to the other groups. **B)**. **absolute vessel number**: no significance was found between the control group (212 /cross section), the DMOG-1 group (229 /cross section) and DMOG-2 group (272/ cross section), but a trend could be observed that the DMOG-1 group tend to have more vessels than the control group, whereas the DMOG-2 group possessed the most vessels. **C)**. **vessel density in cross section**: The DMOG-2 group significantly possessed a higher vessel density (3.0 x 10^-5^/μm^2^) than the control group (1.2 x 10^-5^/μm^2^, *p < 0.05) and the DMOG-1 group (1.6 x 10^-5^/μm^2^ #p < 0.05).

Comparing to the other groups, the DMOG-2 group possessed a significantly smaller cross section area. (17.8 × 10^6^μm^2^, 14.4 × 10^6^μm^2^ versus 9.4 × 10^6^μm^2^, *^,#^ p < 0.05). The cross section area of the control group was bigger than the experimental groups (Figure 5A).

As for the absolute vessel number, no significance was found between the control group (212/cross section), the DMOG-1 group (229/cross section) and DMOG-2 group (272/cross section), but a trend could be observed that the DMOG-1 group tend to have more vessels than the control group, whereas the DMOG-2 group possessed the most vessels (Figure 5B).

The DMOG-2 group significantly possessed a higher vessel density (3.0 × 10^-5^/μm^2^)than the control group (1.2 × 10^-5^/μm^2^, *p < 0.05) and the DMOG-1 group (1.6 × 10^-5^/μm^2^ , #p < 0.05) (Figure 5c).

### DMOG effects on cell proliferation in the AV loop: Ki67 IHC: more positive cells in DMOG-2 group

**Figure 6:**
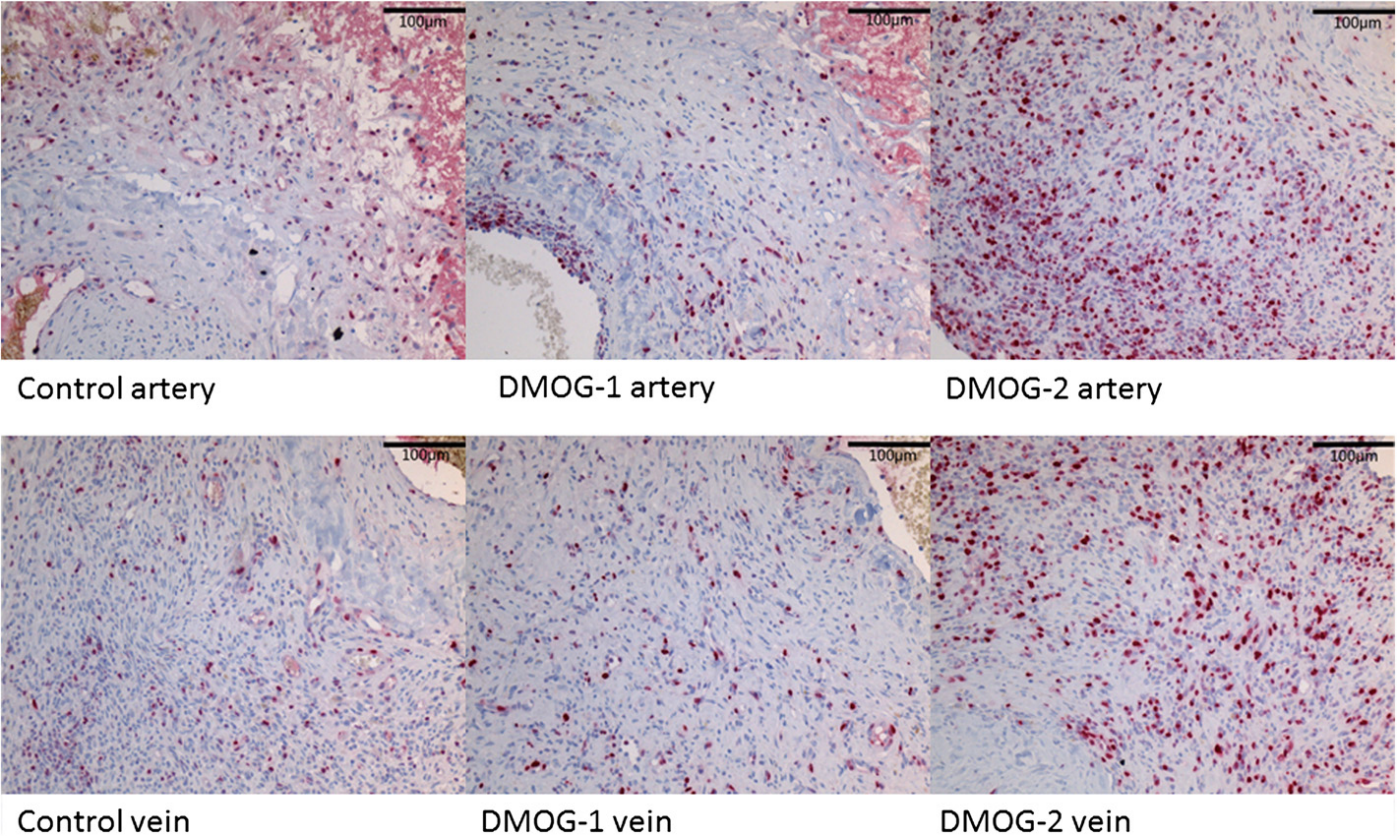
**Ki67 Immunohistochemistry staining.** The slides were picked from the central transversal section, and stained for Ki67 which was a proliferation indicator located in the nuclei. Positive cells were rosa red in nuclei while the negative cells were blue. Pictures from the central artery and vein were taken for each group. A few positive cells could be found in the control group and DMOG-1 group, and tremendous positive cells could be found in the DMOG-2 group, indicating that in the DMOG-2 group the proliferation of the cells were more active.

Ki67 is a nucleus protein that is associated with cell proliferation activity [30]. In Ki67 IHC staining, positive cells were red in nuclei whereas the negative cells counter stained with haematoxylin were with blue nuclei. More positive cells were observed in DMOG-2 group all over the slide, (Figure 6: control group artery and vein/DMOG-1 group artery and vein/DMOG-2 group artery and vein) indicating that DMOG may not result in the depression of proliferation of cells in vivo.

### DMOG effects on angiogenic gene expression levels in the AV loop: real time PCR: mRNA of HIF-1α and its angiogenic target genes increased

**Figure 7:**
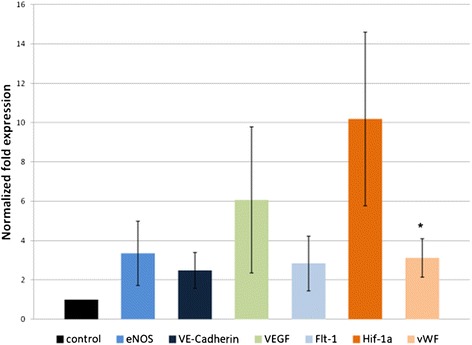
**mRNA normalized fold expression.** The DMOG-2 group was given DMOG in 15 mg/Kg by i.p. injection at day 8, day10 and day 12 (n = 6). The specimens were explanted at day 14 and the distal 2/5 part of the clots were sent to mRNA analysis. Data given are mean mRNA levels of HIF-1α, eNOs, VE cadherin, VEGF, Flt-1, and vWF expressed as fold increase above control group (*P < 0.05).

The distal 2/5 part of the fibrin clot containing the AV loop as the vascular outgrowth from the axial loop was sent to RNA isolation, the mRNA of HIF-1α as well as its target genes that were considered to be related to angiogenesis, including: eNOs, VE cadherin, VEGF, Flt-1, and vWF were quantified by real time PCR [31]. The graph below demonstrates the mRNA levels of DMOG-2 group compared to the control group in folds. The HIF-1α elevated more than 10 folds in the DMOG-2 group. Correspondingly, its target genes elevated to different extends. In particular the vWF raised significantly (*P < 0.05) (Figure 7).

## Discussion

The AV loop model is a promising vascularization model in the in vivo tissue engineering studies. The loop underwent hypoxia in the isolation chamber and the hypoxia pattern may have some relationship with the vascularization process according to our previous study which investigated the hypoxia pattern in the AV Loop spatially and temporally. The present study intends to prove, that the PHD inhibitor DMOG could be able to stimulate the vessel outgrowth from the axial AV loop when it stabilizes HIF, hence to prove that HIF-1α is the stimulus for vascularization in the AV loop model. Beside effects of hypoxia angiogenesis and vasculogenesis can also be a mechanically induced phenomenon from effects of turbulences and shear stress [11]. Mechanical forces themselves can play an important role through shear stress in producing a subcritical ischemic area where angiogenesis and vasculogenesis must start.

### PHD inhibitor up-regulate HIF-α

The PHD inhibitor has been used as HIF-α stabilizer to suppress the HIF-α degradation by hydroxylase pathway in studies that needs to up-regulate HIF-α. Warnecke et al had successfully induced HIF-α expression in vivo and in vitro. The renal HIF-α was increased and by repeated local injection into the sponge that was embedded subcutaneously, angiogenesis was induced [32]. Song et al had dissolved DMOG in PBS and delivered DMOG to rats in 15 mg/kg/day via an osmotic pump and had a renoprotection and HIF-α induction accompanied by a reduction of oxidative stress, inflammation and fibrosis [25]. Nagel et al discovered that DMOG given to rats at 0 h,8 h and 16 h postoperatively in 40 mg/kg by i.p. injection helped to improve the behavior after brain ischemia [24]. This study proved that in the early stage (upon 2 weeks of implantation) vascularization process in the AV loop could be accelerated by the PHD inhibitor DMOG, with smaller dosages maintained in the system, accompanied with the HIF-1α protein increase.

### Kinetics of DMOG in rat

DMOG given at lower dosage constantly after one week tend to have better effect than the group given at larger dosage in the early stage in this model. Till now the kinetics in the rat is still unclear, either from the manufacturer or from the literature. What we do know is that according to the molecular structure provided by the manufacturer (Figure 1), this chemical possesses strong polarity, which means it is highly soluble in water. It may not be active in solution for over one week, and it chelates with Fe (II) rapidly and strongly.

Since the fibrin matrix is porous [33] and proved to be able to function as slow release system for growth factors such as VEGF and bFGF [34], it may be also interesting that whether DMOG can be loaded in the matrix upon the implantation of the AV Loop, and be released later on. In the preliminary experiment the DMOG was dissolved in fibrin in different dilution in Teflon chambers and imbedded in as up-side-down manner as described before [35] and the dorsal muscle that attach to the fibrin clot were examined at day 7 and day 1, and there was no sign of HIF-1α up-regulation or increase of vascularization (data not shown).

Subsequently the DMOG was applied systemically by i.p. injection. As the Kidneys were sensitive to DMOG, [32] [25] it was utilized as organ control to indicate the systemic level of drug. In this study, kidneys from both of the control group and the DMOG-1 group were negative whereas in DMOG-2 group positive cells were observed, indicating that DMOG would be degraded or expelled from the system within days, and won’t maintain for more than a week. From this we can infer that administrate the drug continuously or repeatedly, rather than in a shock therapy would maintain this drug in system as long as possible. The time points of applying the drug are also important in this study. According to the previous investigation for the pattern of vascularization of the AV Loop, there are few cells exudate from the central vessels at the first week upon the loop implantation. The granulation tissue which contains newly grown vessels generate rapidly in the second week when tremendous cells could be observed in the fibrin clot [5]. So in the second week there are much more cells that can be induced to accumulate more HIF-1α, and hence more tremendous outcome in vessel outgrowth.

### Further effects of PHD

As the PHD family inhibit HIF-α via competitively chelating the Fe (II), which is an essential co-factor for many enzymes [36], they may have effects in diverse physiological activities which those enzymes are involved in. In Song’s study the DMOG treatment decreased macrophage infiltration and reduced fibrosis [27]. However, in our study, the Ki67 IHC which indicates the cell proliferation [30] demonstrates that DMOG does not inhibit the proliferation but on contrary activate the mitosis of the infiltrating cells in the fibrin matrix.

In this study, the HIF-1α was up-regulated at protein level, but also at the mRNA level, indicating that DMOG may be also involved in the HIF-α up-regulation transcriptionally, aside from stabilizing the protein from hydroxylation.

### Perspectives

In order to generate a three dimensional construct, it seems necessary that cultured cells are seeded into a scaffold that can mimic the structure and the environment for cell growth, and allow the exchange of oxygen, nutrient and the waste. As an in vivo animal model for tissue engineering, specific cells can be seeded in the chamber to generate corresponding tissue or organ [9,10]. As an in vivo model, the knowledge about the vascularization pattern corresponding to hypoxia and HIF-1α expression can help modulate the vascular growth. Since bFGF and VEGF immobilized in the fibrin matrix can improve the vascularization of AV loop [34] and it may be a superior choice to manipulate the neo vessel outgrowth by up-regulation their transcription factor HIF-1α [28], the PHD inhibitor is a good option.

### Summary

The PHD inhibitor DMOG was utilized as HIF-1α stablizer in the study. DMOG was applied in two ways: at early stage (day 0, day3) of the loop in larger dosage (40 mg/kg), or at later stage (day 8, day 10, day 12) in smaller dosage (15 mg/kg), namely DMOG-1 group and DMOG-2 group. Upon the explantation at day 14, DMOG was only still functioning in the system in DMOG-2 group. This was evidence by the HIF-1α IHC for the kidneys that were sensitive to the PHDs stimulation that HIF-1α positive renal cells were only observed in DMOG-2 group.

Also, in DMOG-2 group the HIF-1α positive cell percentage raised significantly and mRNA was over 10 folds over the control group, indicated that HIF-1α was up-regulated at protein level and mRNA level. The mRNA of its angiogenic target genes such as eNOs, VE cadherin, VEGF, Flt-1, and vWF were up-regulated as well. The cross section area decreased and the vessel denstiy increased in DMOG-2 group, hint that the fibrin clot was degraded faster and the vascularization more active.

Addistional evidence was the Ki67 IHC which could demonstrate the activuty of cell proliferation. In DMOG-2 group there were tremendous Ki67 positive cells comparing to the control group and the DMOG-1 group.

## Conclusions

In summary the process of the vascularization can be stimulated by DMOG in the AV Loop model. DMOG given at lower dosage constantly after one week tends to have better effect than the group given at larger dosage in the early stage in this model, and promotes the cell proliferation.
